# The impact of NaI(Tl) crystal hydration on gamma camera spectral response and image uniformity

**DOI:** 10.1002/acm2.70218

**Published:** 2025-08-21

**Authors:** Grace Eliason, Michael S Silosky

**Affiliations:** ^1^ Department of Radiology University of Colorado School of Medicine Aurora, Colarado USA

**Keywords:** gamma camera, hydration, NaI(Tl), nuclear medicine, QC

## Abstract

**Background:**

NaI(Tl) scintillators used in most gamma cameras are hermetically sealed to prevent the absorption of water molecules from the surrounding environment. If this seal is compromised, crystal hydration, a localized defect resulting in non‐uniform attenuation of scintillation photons, may occur.

**Purpose:**

The purpose of this study was to evaluate the effects of crystal hydration across multiple radionuclides, characterizing the impact on spectral response and image uniformity.

**Methods:**

Hydration was assessed using on‐peak and off‐peak imaging for several radionuclides. The progression of hydration was assessed by calculating the contrast‐to‐noise ratio in hydrated regions on monthly uniformity maps. Spectra were captured for both hydrated and non‐hydrated regions of the detector to determine photopeak energy and energy resolution.

**Results:**

The visual appearance of the hydration effect in off‐peak images was greatest for ^133^Xe. The effect was more substantial in the 10% low off‐peak image than in the 10% high off‐peak image. Hydration was not observed in on‐peak images for either ^133^Xe or ^99m^Tc and was only barely visible for ^131^I. CNR measurements show a slow but notable progression of hydration in uniformity maps over time. The measured photopeak in hydrated regions was lower than that of non‐hydrated regions. Hydration also resulted in a degradation in energy resolution with the effect being more significant at lower energies.

**Conclusions:**

Hydrated regions of the detector demonstrated an energy‐dependent degradation in energy resolution which corresponded to an energy dependence in the detectability of hydration in off‐peak images. Monthly updates to the uniformity correction maps were able to adequately account for hydration. When hydration has been identified, routine evaluation of its impact on uniformity maps may provide a convenient method of tracking progression.

## 1 INTRODUCTION

Despite advances in solid state detectors, thallium‐activated sodium iodide (NaI(Tl)) scintillators remain the most common detection medium used in conventional gamma cameras. Because NaI(Tl) is hygroscopic, that is, it attracts and absorbs water from the surrounding environment, NaI(Tl) scintillators used in gamma cameras are hermetically sealed. In cases where the seal is imperfect, it is possible for these detectors to absorb water molecules from the surrounding air, resulting in a specific defect known as crystal hydration.^1^, ^2^ The consequence of this defect is a non‐uniform increase in the reabsorption of scintillation photons within the detection medium, typically near the detector surface. In hydrated regions of the scintillation crystal, the average number of scintillation photons interacting with the photocathodes of the photomultiplier tube array is reduced. Ultimately, this results in an apparent shift of the photopeak in these regions.^1^, ^2^ Under normal imaging conditions, the photopeak is centered within an energy window which determines which detection events will be included when images are generated. The shift in the observed photopeak in hydrated regions may result in a localized reduction in signal under these conditions. Given the deleterious effects of crystal hydration on image uniformity, multiple quality assurance (QA) guidelines recommend tests specifically designed to identify crystal hydration.^3^, ^4^, ^5^ These tests include off‐peak imaging, which accentuates the effects of hydration related non‐uniformities, allowing for earlier detection. Because the average energy of detection events in hydrated regions will be reduced relative to that observed by the detector as a whole, energy windows centered below the photopeak will undergo a small but detectable increase in signal in these regions relative to the rest of the imaging field of view (FOV). Further, energy windows centered above the photopeak will have the opposite effect with a reduction in signal in hydrated regions. This phenomenon provides a means of detecting crystal hydration before it has a substantial effect on image quality. Unfortunately, the process of hydration is not “reversible” and detectors with serious hydration issues cannot be repaired.^3^, ^4^, ^5^ If crystal hydration is identified, it becomes necessary to determine if the effects are significant enough to warrant detector replacement. However, the decision may have substantial consequences both financially and in terms of clinical care. Further, while guidance exists regarding the detection of crystal hydration, there is little information regarding quantification of its effect. The purpose of this study was to evaluate the effects of crystal hydration, including an assessment of hydration utilizing off‐peak imaging, an evaluation of hydration effects on image uniformity for various radionuclides, and a characterization of the effects of hydration on observed photopeak and energy resolution.

## 2 METHODS

**FIGURE 1:**
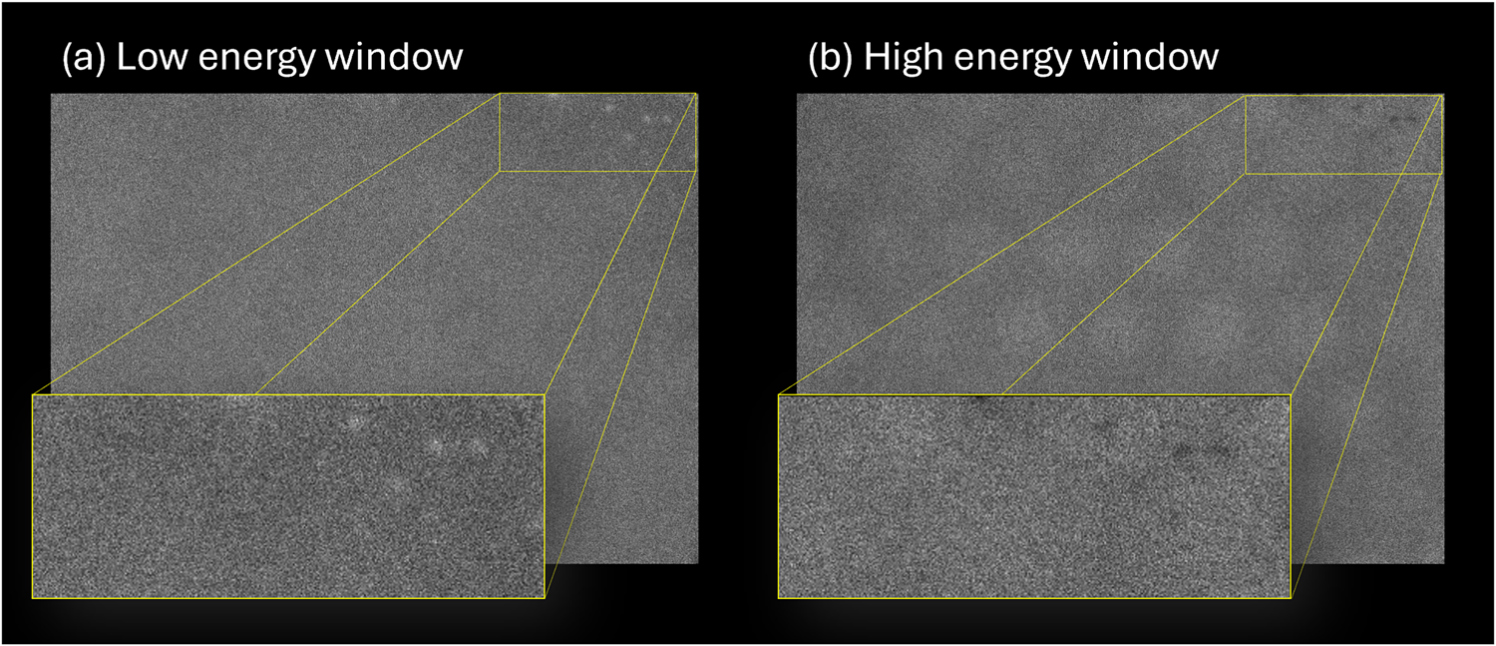
Resulting images from hydration tests performed during the annual physics evaluation of a gamma camera. Hydration was noted in the upper right corner of detector 1, as shown in the insert.

All images and spectra were acquired on a dual‐head Symbia T16 single‐photon emission computed tomography (SPECT) / computed tomography (CT) system (Siemens Medical Solutions, Malvern, PA) with a 3/8‐inch thick NaI(Tl) crystal. A “measles‐like” pattern indicating crystal hydration was identified in the upper‐right corner of detector 1 during the unit's annual physics evaluation, as shown in Figure 1. All acquisitions were captured using a 1024 × 1024 matrix, zoom factor of 1, and collimators removed. All data was acquired in December 2024 with the exception of the monthly calibration floods, which were acquired over a period of several years.

### 2.1 Hydration and uniformity floods

**FIGURE 2:**
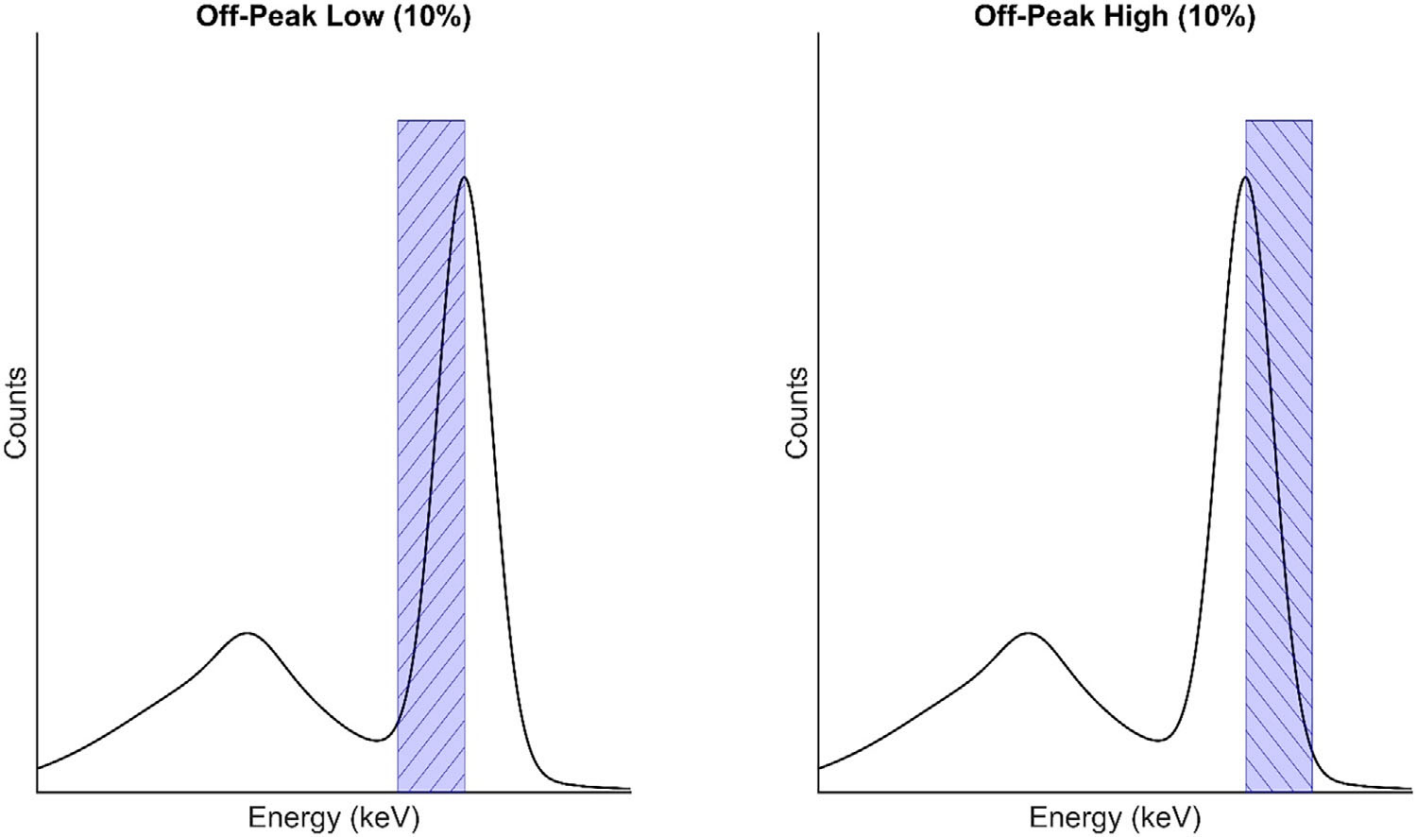
Example spectra of asymmetric energy windows used to identify hydration on clinical gamma cameras. Low‐ and high off‐peak floods were acquired at window widths of 10% symmetrically about the photopeak.

To assess the state of crystal hydration, off‐peak intrinsic floods were acquired as per IAEA recommendations.^5^ High‐ and low‐energy off‐peak windows (10% width) positioned above and below photopeak were acquired, as shown in Figure 2. These windows were fixed adjacent to a 1% window centered about the photopeak. This allowed the camera to properly center the entire energy window group on the observed photopeak. These tests were performed using xenon‐133 (^133^Xe), technetium‐99m (^99m^Tc), and iodine‐131 (^131^I), which were selected for their nominal photopeaks of 81 keV, 140 keV, and 364 keV,^6^ respectively. Point sources were placed centrally at a distance of five FOV away from both detectors.

On‐peak intrinsic floods were acquired under the same conditions as described above using vendor default photopeaks and energy windows for ^133^Xe (80 keV, 20%), ^99m^Tc (140 keV, 15%), and ^131^I (364 keV, 20%) to assess the impact of crystal hydration on routine uniformity tests. Stopping conditions of 30,000 kilocounts were used as per NEMA recommendations.^7^ The vendor of the gamma camera in question recommends 200,000 kilocount monthly intrinsic calibration floods as part of routine quality control (QC). These floods are used to create uniformity correction maps, which were applied to all images. Additionally, all uniformity images were acquired in the same setup and session as the off‐peak floods.

**FIGURE 3:**
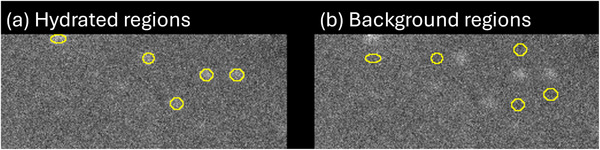
Five ROIs surrounding (a) hydrated and (b) background regions in the hydrated corner for a ^133^Xe low‐energy off‐peak image. Regions treated as single hydrated and background ROIs for analysis.

Off‐ and on‐peak flood images were visually inspected for non‐uniformities with a particular attention to a “measles‐like” pattern.^3^, ^4^, ^5^ Hydration was identified in five primary locations in the upper right corner of detector 1. To quantify the magnitude of hydration, regions of interest (ROIs) were drawn manually in ImageJ (version 1.54, National Institutes of Health, Bethesda, MD) around these locations with paired background regions of the same size drawn and placed slightly off‐set from the hydrated regions, as shown in Figure 3. The five individual hydrated regions were treated as a single ROI, as were the five background regions. The mean (μ) and standard deviation (σ) of the signal from the hydrated and background regions were recorded and used to calculate the contrast‐to‐noise ratio (CNR) as:

$$\mathrm{CN}R_{\mathrm{hydration}}=\frac{\mu_{\mathrm{hydration}}-\mu_{\mathrm{background}}}{\sigma_{\mathrm{background}}}$$

The $\mathrm{CN}R_{\mathrm{hydration}}$ was calculated for the 10% low, 1% center, and 10% high off‐peak floods for ^133^Xe, ^99m^Tc, and ^131^I. An increase in $\mathrm{CN}R_{\mathrm{hydration}}$ was assumed to correspond to the progression of crystal hydration.

### 2.2 Monthly calibration floods

Monthly 200,000 kilocount intrinsic calibration floods were acquired as per vendor recommendations using a Co‐57 point source and the vendor's automated QC functionality. At the time of these experiments, the hydration in the upper right corner of detector 1 was visible in the monthly calibration flood, affecting the uniformity correction being applied to all clinical images. The same hydration and background ROIs described above were applied to the monthly calibration floods acquired between March 2020 to March 2025 to assess the progression of hydration over time. Matching ROIs were placed in the upper left non‐hydrated corner of detector 1 for comparison. CNR for both corners was calculated for all calibrations performed during this date range, barring some intermittent months of missing data. In addition, differences between the two detectors and longitudinal trends were assessed using the scanner‐reported integral and differential uniformity values for the useful and central FOVs as defined in NEMA NU 1–2023.^7^

### 2.3 Spectral experiments

**FIGURE 4:**
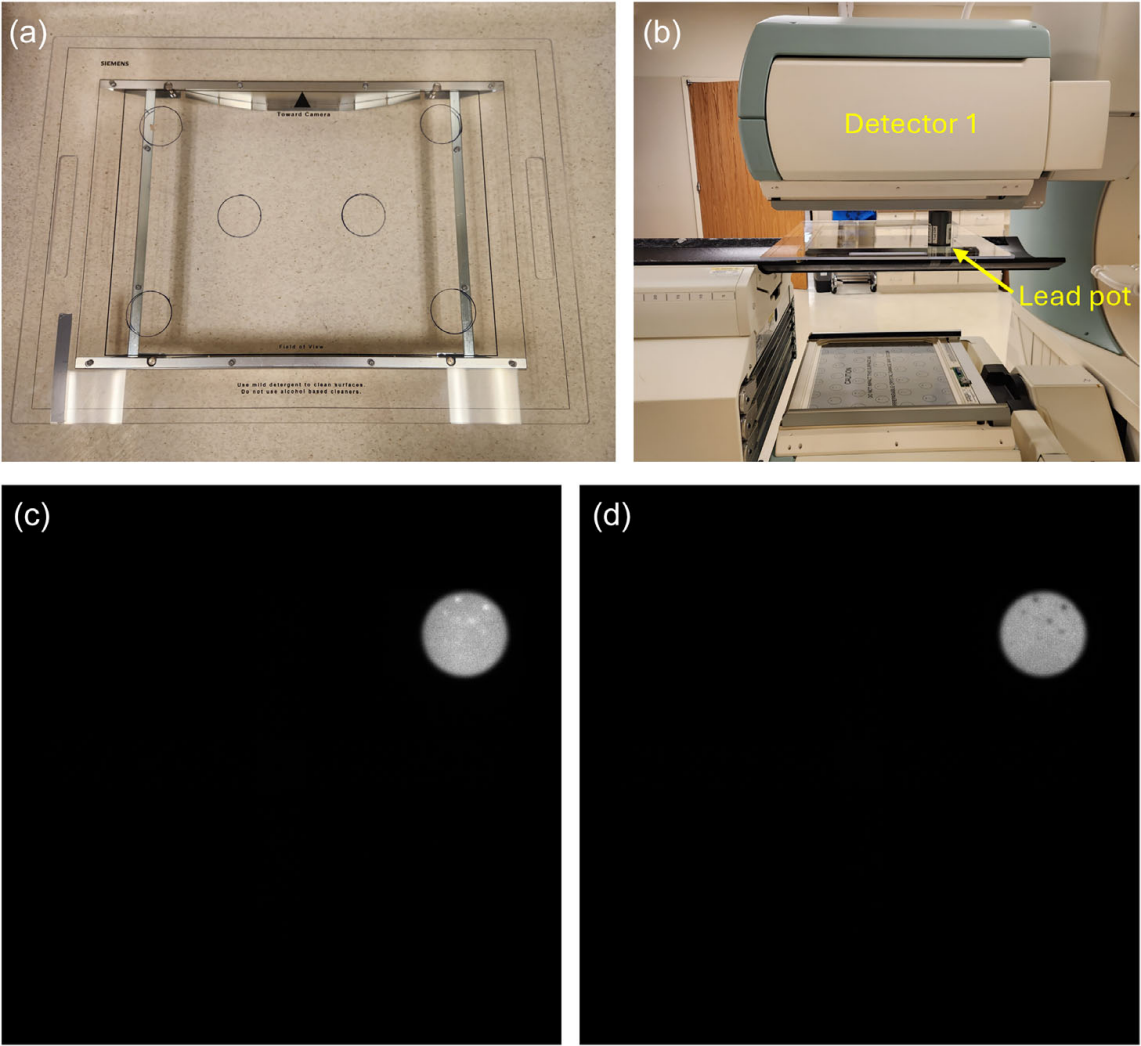
Experimental setup, including (a) top‐down view of source placement device, (b) side‐view of relative positioning with source in lead pot, and resulting (c) low‐and (d) high‐energy window images of exposed hydrated region of detector.

To compare the spectral response of hydrated versus non‐hydrated regions of the detector, sources were placed in a lead pot and used to expose six different regions of the detector face as pictured in Figure 4a. The upper right corner of the FOV was the only region found to exhibit hydration. Corner and center regions were independently characterized to eliminate possible edge effects on obtained spectra. Table and detector heights were chosen based upon sufficient coverage of the hydrated region, as shown in Figure 4b and c. The radionuclides used in this experiment were ^133^Xe, ^99m^Tc, indium‐111 (^111^In), and ^131^I, which were selected for their range of photopeak energies, with ^111^In having nominal photopeaks at 172 and 247 keV.^6^ Five spectra were collected in each of the six positions for each radionuclide to account for statistical variations using the system's analyzer function. Spectra were collected using the scanner's standard protocol.

Each spectrum was characterized using a single‐term Gaussian with constant offset and bounds of ± 3 standard deviations from the nominal photopeak to eliminate contributions from nearby peaks:

$$F\;\left(x\right)=ae^{-\frac{1}{2}{\left(\frac{x-b}{c}\right)}^{2}}+d,$$

where d is the constant offset. From this, the photopeak and energy resolution were obtained as:

$$\begin{array}{c} \mathrm{Fitted}\;\mathrm{photopeak}\;=b\;\mathrm{and}\;\mathrm{Fitted}\;\mathrm{energy}\;\mathrm{resolution}\;\\ =\frac{\mathrm{FWHM}}{E_{\mathrm{peak}}}\;\times 100=\frac{2\sqrt{2\ln2}\;c}{b}\;\times 100, \end{array}$$

respectively. All fits were performed using MATLAB (R2023b, Mathworks Inc., Natick, MA).

To determine if the photopeak and energy resolution were statistically different between regions, a two‐tailed t‐test for distributions with unequal variances was used. Resulting *p*‐values of less than 0.05 were considered statistically significant. Additionally, the shift in photopeak between regions was calculated as a percentage of the nominal photopeak.

## 3 RESULTS

### 3.1 Hydration and uniformity floods

**FIGURE 5:**
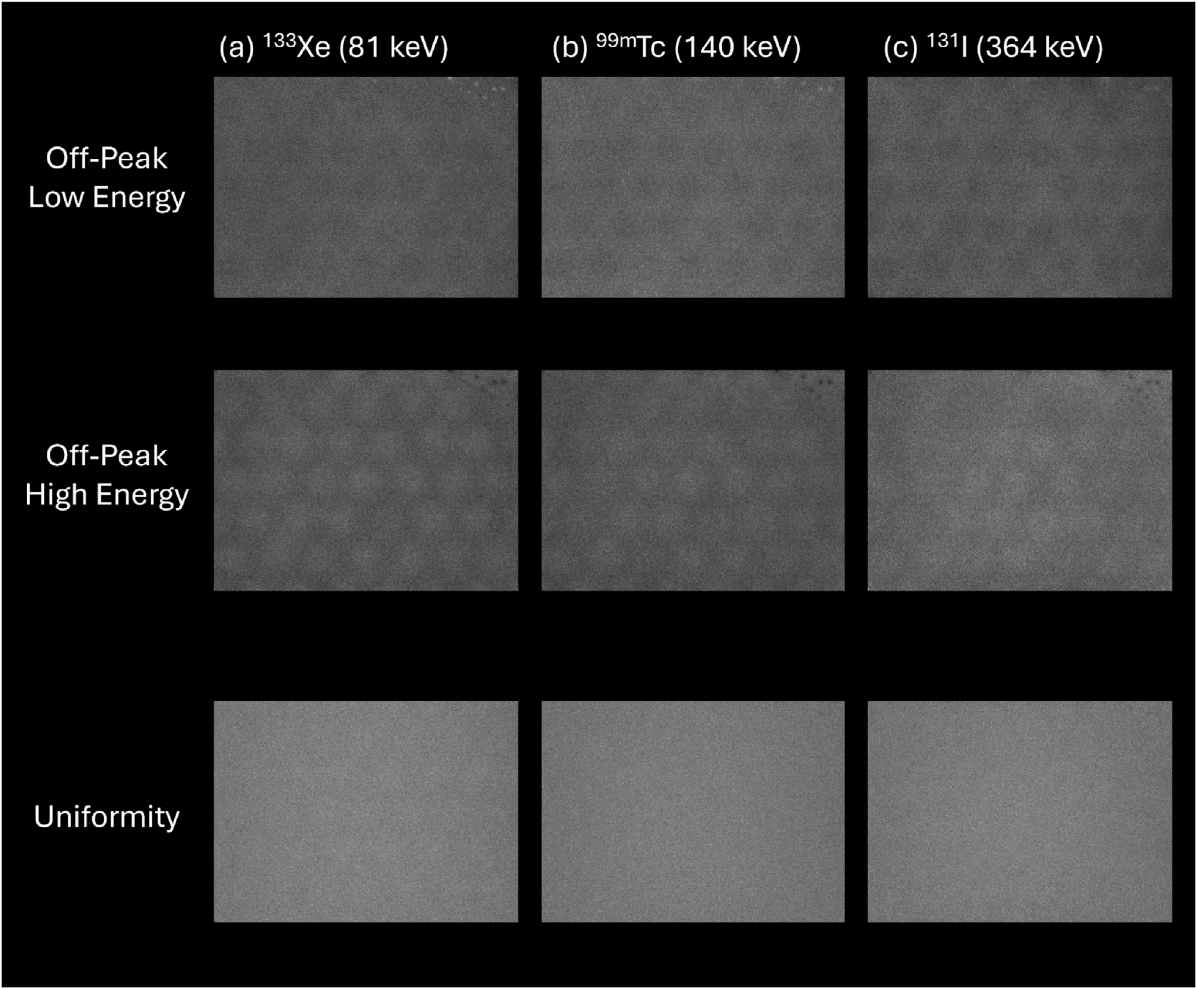
Intrinsic hydration and uniformity floods for (a) ^133^Xe, (b) ^99m^Tc, and (c) ^131^I for a clinical gamma camera detector exhibiting hydration. Off‐peak hydration floods captured using asymmetric 10% energy windows, uniformity floods captured using vendor default energy windows for each radionuclide. Brighter spots indicate “hotter” or higher signal, and darker spots indicate “colder” or lower signal.

Off‐peak intrinsic floods for ^133^Xe, ^99m^Tc, and ^131^I for the hydrated detector are shown in Figure 5. Hydration was noted as a collection of small “spots” or subregions in the upper right corner of the imaging FOV. Signal in hydrated subregions appeared brighter than background on low off‐peak floods and darker on high off‐peak floods. Differences between high and low off‐peak floods were more visually apparent for radionuclides with lower energy photopeaks.

On‐peak intrinsic, or uniformity, floods for ^133^Xe, ^99m^Tc, and ^131^I are also shown in Figure 5. Hydration was not visible to the observer in the ^133^Xe and ^99m^Tc scans and is barely visible in the ^131^I scan.

Mean signal, standard deviation, and CNR from hydrated and background ROIs off‐peak images are provided in Table 1. In the low energy off‐peak floods, CNR of the hydration decreased with increasing radionuclide energy; in the high energy floods; however, CNR remained somewhat constant. This was corroborated by the visual differences seen between radionuclides in Figure 5. Uniformity floods revealed no clear trend or relationship with photopeak energy, nor was hydration visible, as seen in Figure 5.

**TABLE 1:** Mean (μ), standard deviation (σ), and CNR of hydrated and background regions for off‐peak flood tests, including both 10% asymmetric and 1% centered window results.

		10% Low	1% Center	10% High
^133^Xe	μ_hyd_ (σ_hyd_)	30.11 (6.35)	21.95 (5.26)	17.82 (4.52)
	μ_bkg_ (σ_bkg_)	22.69 (4.93)	22.00 (4.91)	24.01 (5.32)
	CNR_hyd_	1.51	−0.01	−1.16
^99m^Tc	μ_hyd_ (σ_hyd_)	30.55 (5.99)	22.12 (5.06)	18.37 (4.51)
	μ_bkg_ (σ_bkg_)	25.61 (5.31)	22.27 (5.00)	24.56 (5.07)
	CNR_hyd_	0.93	−0.03	−1.22
^131^I	μ_hyd_ (σ_hyd_)	23.63 (5.44)	20.56 (4.73)	21.50 (5.01)
	μ_bkg_ (σ_bkg_)	20.06 (4.59)	19.99 (4.38)	27.43 (5.13)
	CNR_hyd_	0.78	0.13	−1.16

### 3.2 Monthly calibration floods

**FIGURE 6:**
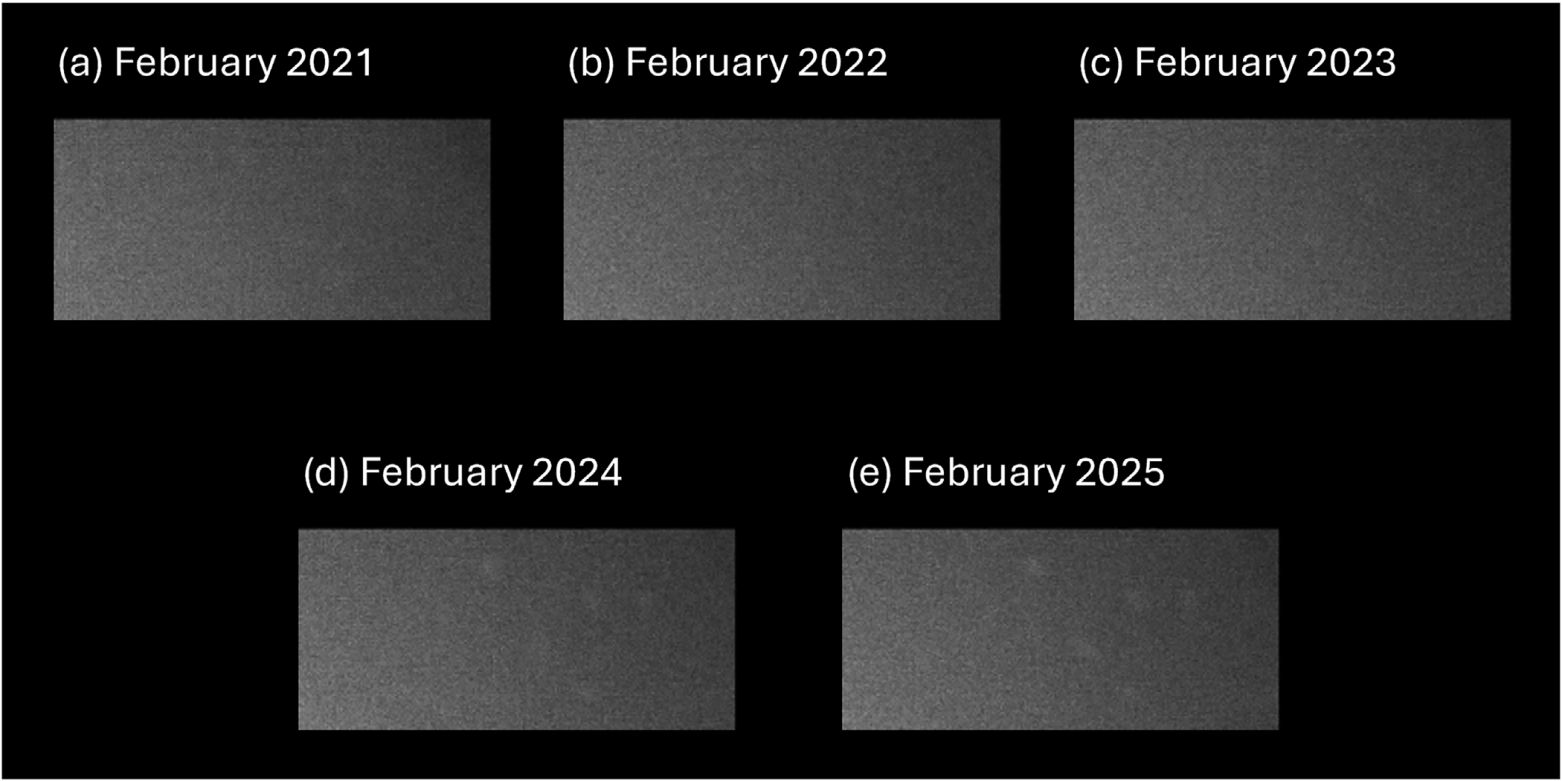
Hydrated corner from monthly calibration floods on detector 1 from February 2021 to 2025. The hydrated region in the upper right corner has become more visualizable over time.

**FIGURE 7:**
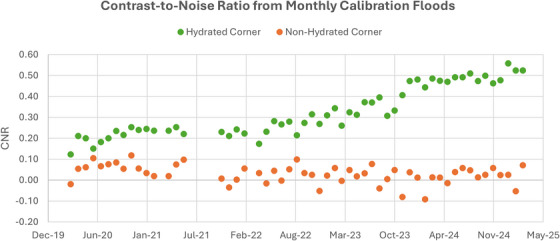
Longitudinal CNR of the hydration from monthly calibration floods for the hydrated corner as compared to the non‐hydrated corner.

Examples of hydration in the upper right corner of the FOV from monthly calibration floods between 2021 and 2025 are provided in Figure 6. Hydration became substantially more noticeable as time progressed, as demonstrated by the five‐fold increase in CNR between December 2020 and March 2025 for the hydrated corner as shown in Figure 7. The non‐hydrated corner remained relatively stable over the same period.

Scanner‐reported integral and differential uniformity values for the useful and central FOVs did not reveal a statistically significant difference between detectors 1 and 2, nor did they reveal any significant trends over time.

### 3.3 Spectral experiments

The center and corner regions without hydration and hydrated region spectra were grouped independently for comparison. The mean and standard deviation of the photopeak and energy resolution for each radionuclide and photopeak energy are provided in Tables 2 and 3, respectively.

**TABLE 2:** For each radionuclide and nominal photopeak energy, the resulting mean (μ) and standard deviation (σ) of the fitted photopeak.

Radionuclide	Nominal photopeak (keV)	Center		Corners without hydration		Hydrated corner	
		μ	σ	μ	σ	μ	σ
^133^Xe	81	80.20	0.02	80.29	0.02	80.20	0.02
^99m^Tc	140	140.38	0.02	140.51	0.02	140.32	0.02
^111^In	172	170.01	0.12	169.86	0.10	169.73	0.01
^111^In	247	244.66	0.16	244.47	0.15	244.30	0.02
^131^I	364	365.15	0.08	365.38	0.09	365.06	0.03

**TABLE 3:** For each radionuclide and nominal photopeak energy, the resulting mean (μ) and standard deviation (σ) of the energy resolution.

Radionuclide	Nominal Photopeak (keV)	Center		Corners without hydration		Hydrated corner	
		μ	σ	μ	σ	μ	σ
^133^Xe	81	10.48	0.05	10.74	0.05	11.09	0.04
^99m^Tc	140	9.23	0.04	9.42	0.05	9.69	0.04
^111^In	172	9.25	0.04	9.43	0.04	9.59	0.05
^111^In	247	9.26	0.03	9.36	0.03	9.47	0.04
^131^I	364	9.06	0.02	9.05	0.02	9.13	0.01

For all radionuclides tested, there was a statistically significant difference in the photopeak between the center and corners (*p* < 0.01). There was also a statistically significant difference between the energy resolution in the corners versus the center (*p* < 0.001), except in the case of ^131^I (*p* = 0.277). In addition, there was a statistically significant difference between the non‐hydrated corners and the hydrated corner in terms of both photopeak (*p* < 0.001) and energy resolution (*p* < 0.01).

**FIGURE 8:**
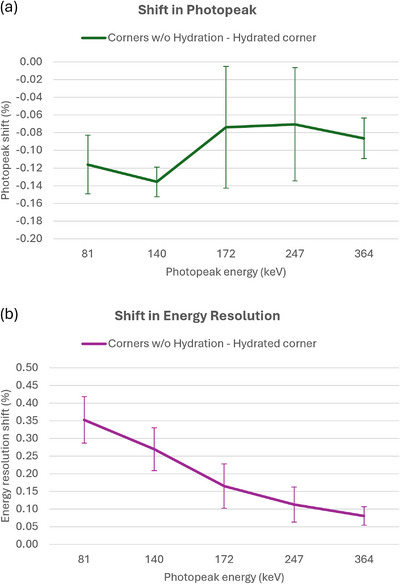
Shifts in photopeak and energy resolution between non‐hydrated corners and hydrated corner of the detector. No trend with energy was found for the shift in photopeak, however, energy resolution was found to decrease for increasing energy.

Due to observed variations between the center and corners, the hydrated corner was compared to the other corners collectively for relative photopeak and energy resolution shift. For the hydrated region, the photopeak shift normalized to the nominal photopeak energy was relatively constant and negative over all photopeak emissions tested, with an average shift of ‐0.10%, as demonstrated in Figure 8a. Additionally, broadening of the photopeak in the hydrated region, as measured by energy resolution, occurred for all radionuclides tested and appears to be energy dependent with lower energy photopeaks more substantially affected, as demonstrated in Figure 8b.

## 4 DISCUSSION

One objective of this study was to evaluate the effects of crystal hydration for various radionuclides. The ability to detect crystal hydration via off‐peak imaging appears to depend on the nominal photopeak energy, as illustrated in Figure 5. In particular, CNR values calculated for the hydrated regions of the 10% low off‐peak images are notably greater for 81 keV emission of ^133^Xe than it is for ^99m^Tc or ^131^I. This is further supported by Figure 8, where the relative broadening of the observed photopeak is more substantial for lower energies even when the relative shift in the photopeak is generally independent of energy. These differences, while statistically significant, are subtle and on the same order of magnitude as regional variations shown in Tables 2 and 3. Additionally, the 10% low energy off‐peak images are more affected than the 10% high off‐peak images. This agrees with a similar observation by Keszthelyi‐Landori, that hydration may be more apparent in low off‐peak images.^2^ Current recommendations^3^, ^4^, ^5^ suggest the use of ^99m^Tc as an appropriate radionuclide when testing for crystal hydration and while this practice is reasonable, radionuclides with lower energy emissions such as ^133^Xe or ^201^Tl are likely to be more sensitive to hydration effects, especially in the low energy off‐peak images.

Crystal hydration did not substantially impact image uniformity for either ^133^Xe or ^99m^Tc on‐peak flood images and only affected ^131^I images quite subtly, as shown in Figure 5. The uniformity corrections, updated on a monthly basis as part of vendor recommended QC, appear to account for these effects in this specific case. However, this may not hold true when hydration is progressing rapidly and uniformity correction generated only weeks earlier become invalid before their next update. In cases where hydration has been identified, an evaluation of the impact of hydration on uniformity corrections, as shown in Figure 7, may be an effective method for evaluating the progression of deleterious effects. An evaluation of integral and differential uniformity values for 200,000 kilocount correction floods was not sufficiently sensitive to detect hydration effects. Consequently, an analysis similar to the targeted CNR analysis performed here is recommended for longitudinal evaluations. Notably, the data represented in Figure 7 indicate changing conditions in the hydrated regions occurring years before the off‐peak hydration test was able to clearly identify the problem.

Arguably, the greatest challenge in dealing with a detector with crystal hydration is determining when replacement is necessary. Ideally, the location and magnitude of the effect of hydration in clinical images would be considered in conjunction with image characteristics from commonly performed clinical exams to determine the relative impact of the defect. However, this type of analysis can be intense in terms of both time and resources. Guidance from the AAPM Task Group 177 Report simply suggests that for any hydration defects, the detector should be scheduled for an evaluation by service and possible replacement.^4^ In practice, replacement of this type may be impractical for a number of reasons, including the impact on the clinical schedule and nature of the service contract. A potential alternative to detector replacement may consist of long‐term monitoring of the camera's ability to address hydration non‐uniformities by applying routine uniformity correction. In cases where these corrections are effective and the state of the detector is relatively stable, it may be reasonable to forgo replacement for an extended period of time without significant impact on clinical images.

While the work described in this study shows promise in both the characterization of the effects of crystal hydration for NaI(Tl) gamma cameras, as well as proposing a means for tracking these effects over time, there are some substantial limitations. First, all evaluations were performed on a single detector which naturally limits the camera model and vendors investigated. As such, additional work on other hydrated detectors is necessary to establish the generalizability of the reported results. This may be challenging as crystal hydration is a relatively uncommon defect among modern gamma cameras. Another limitation is that all spectral data were acquired at the same time. Consequently, the relationship between the observed photopeak and energy resolution for the emissions from different radionuclides was specific to the state of the detector at that time. As hydration tends to worsen over time, the difference in observed effects between radionuclides may vary as well. Future work repeating these evaluations may help characterize these differences. Finally, the hydration effect was detected in a single corner of the detector in question. As mentioned, regional variations were observed in observed photopeak energy and energy resolution. Whether or not observed patterns in these metrics would remain consistent in more centrally located cases of hydration remains to be determined.

## 5 CONCLUSION

This study evaluated the effects of crystal hydration on image uniformity and spectral response for several radionuclides on a clinical gamma camera. Hydrated regions of the detector demonstrated an energy‐dependent degradation in energy resolution. This corresponded to a greater detectability of these regions when performing off‐peak imaging with lower energy radionuclides. However, routine uniformity calibrations recommended by the manufacturer were sufficient to correct for the effects of crystal hydration, regardless of the radionuclide used for imaging. Additionally, this study has demonstrated a potentially useful approach for tracking the progression of crystal hydration after it has been identified by monitoring the CNR of hydrated regions in high‐count uniformity correction floods.

## AUTHORS CONTRIBUTION

Both authors were responsible for methodology, investigation, resources, and writing. The second author was also responsible for conceptualization, supervision, and project administration.

## CONFLICT OF INTEREST STATEMENT

The authors have no relevant conflicts of interest to disclose.
